# A Double-Blind, Randomized, Crossover Comparative Study for Evaluating the Clinical Safety of Ephedrine Alkaloids-Free Ephedra Herb Extract (EFE)

**DOI:** 10.1155/2018/4625358

**Published:** 2018-08-05

**Authors:** Hiroshi Odaguchi, Mariko Sekine, Sumiko Hyuga, Toshihiko Hanawa, Keika Hoshi, Yoshinobu Sasaki, Masako Aso, Jinwei Yang, Masashi Hyuga, Yoshinori Kobayashi, Takashi Hakamatsuka, Yukihiro Goda, Yuji Kumagai

**Affiliations:** ^1^Oriental Medicine Research Center, Kitasato University, 5-9-1 Shirokane, Minato-ku, Tokyo 108-8642, Japan; ^2^Department of Hygiene, Kitasato University School of Medicine, 1-15-1 Kitasato, Minami-ku, Sagamihara, Kanagawa 252-0374, Japan; ^3^Kitasato University Hospital Clinical Trial Center, 1-51-1 Kitasato, Minami-ku, Sagamihara, Kanagawa 252-0375, Japan; ^4^Tokiwa Phytochemical Co., Ltd., 158 Kinoko, Sakura, Chiba 285-0801, Japan; ^5^National Institute of Health Sciences, 3-25-26 Tonomachi, Kawasaki-ku, Kawasaki, Kanagawa 210-9501, Japan; ^6^Department of Pharmacognosy, Kitasato University School of Pharmacy, 5-9-1 Shirokane, Minato-ku, Tokyo 108-8641, Japan

## Abstract

Ephedra Herb is an important crude drug; it is used in various Traditional Japanese Medicine (Kampo) formulations. Its significant pharmacological effects have been believed to be attributed to ephedrine and pseudoephedrine, which sometimes induce adverse effects. On the other hand, it has been reported that some of these pharmacological effects are not dependent on ephedrine or pseudoephedrine. Ephedrine alkaloids-free Ephedra Herb extract has been newly developed. It has been reported to have analgesic, anti-influenza, and antimetastatic effects. This clinical trial was aimed at verifying the noninferiority of EFE's safety compared to that of Ephedra Herb extract (EHE) in humans. This was a single-institution, double-blinded, randomized, two-drug, two-stage, crossover comparative study. Twelve healthy male subjects were equally and randomly allocated into two groups: prior administration of EFE (EFE-P) and prior administration of EHE (EHE-P). In Stage 1, EFE and EHE were orally administered to the EFE-P and EHE-P groups, respectively, for six days. After a 4-week washout period, Stage 2 was initiated wherein the subjects were given a study drug different from Stage 1 study drug for six days. Eleven adverse events with a causal relationship to the study drugs (EHE: 8; EFE: 3) were noted; all events were mild in severity. With regard to the incidence of adverse events, EHE and EFE administration, respectively, accounted for 4 cases (out of 12 subjects, similarly below) and 1 case of increased pulse rate (p=0.32) and 3 cases and 1 case of insomnia (p=0.59). Further, there was one case of hot flashes (p=1.00) due to EFE administration and one case of dysuria (p=1.00) due to EHE administration. There were no significant differences in the incidences of adverse events between EHE administration and EFE administration. Therefore, we concluded that EFE is not inferior to EHE in terms of safety.

## 1. Introduction

In the 17th edition of the Japanese Pharmacopoeia, Ephedra Herb has been defined as the terrestrial stem of* Ephedra sinica* Stapf.,* Ephedra intermedia* Schrenk et C.A. Meyer, or* Ephedra equisetina *Bunge (Ephedraceae), with an ephedrine alkaloid (ephedrine and pseudoephedrine) content greater than 0.7% [1]. In Japan, Ephedra Herb is used as an ingredient in various Traditional Japanese Medicine (Kampo) formulations (such as eppikajutsuto, kakkonto, kakkontokasenkyusin'i, kakkonkajutsubuto, keishimaokakuhanto, gokoto, goshakusan, shoseiryuto, shimpito, zokumeito, bofutsushosan, maoto, maobushisaishinto, makyokansekito, makyoyokukanto, and yokuininto) for various treatments (http://mpdb.nibiohn.go.jp/stork/). For example, kakkonto is used to treat the common cold in the early stage and stiff neck, and maoto is effective against early flu symptoms and rheumatoid arthritis. Makyoyokukanto alleviates joint, nerve, and muscle pain, while eppikajutsuto has a therapeutic effect on rheumatoid arthritis (http://mpdb.nibiohn.go.jp/stork/).

Ephedra Herb is widely known for its diaphoretic, antipyretic, antitussive, anti-inflammatory, and analgesic effects, and these pharmacological effects are thought to be attributable to ephedrine alkaloids (EAs) [2]. Ephedrine (Eph) and pseudoephedrine (Pse), EAs whose structures are similar to that of adrenaline, stimulate adrenaline receptors and result in expansion of the bronchi and elimination of nasal mucosal hyperemia. Furthermore, the anti-inflammatory effect of Ephedra Herb has been thought to be attributable to Pse [3]]. EAs have also been reported to inhibit prostaglandin E2 biosynthesis [4].

On the other hand, it is known that EAs contained in Ephedra Herb induce hypertension, palpitations, insomnia, dysuria, and other side effects. Consequently, caution is required when Kampo medicines containing Ephedra Herb are used by patients with circulatory impairment, hypertension, or renal impairment; those who are physically fragile; or the elderly. The US Food and Drug Administration has prohibited the sale of all EAs-containing products, such as Ephedra Herb, because of deaths due to excessive intake or inappropriate use of supplements containing Ephedra Herb [5]. Further, Eph and Pse have been proscribed as doping substances; therefore, caution is required when athletes take Kampo medicines containing Ephedra Herb.

We have previously reported that Ephedra Herb extract (EHE) inhibits the phosphorylation of the hepatic growth factor (HGF) receptor, c-Met, thereby suppressing HGF-induced cancer cell motility and metastasis [6–8]. Moreover, we found that the non-alkaloidal fraction of EHE has a c-Met-inhibitory effect. Herbacetin glycosides have also been discovered from the fraction [9], and we found that herbacetin, the aglycone of herbacetin glycosides, has multikinase-inhibitory effects, including a c-Met-inhibitory effect [10]. However, considering that the amount of herbacetin glycosides and their relative activity are low, the c-Met-inhibitory activity of EHE cannot be attributed to herbacetin glycosides alone and multiple other active ingredients are believed to play a role.

The aforementioned results indicate that some of the pharmacological effects of Ephedra Herb are not EA-dependent. Therefore, we used ion-exchange column chromatography to eliminate EAs from EHE to obtain EAs-free Ephedra Herb extract (EFE) [11]. Similar to EHE, EFE has a c-Met-inhibitory effect, as well as analgesic and anti-influenza effects [12]. Thus, EFE could replace Ephedra Herb in clinical applications.

With regard to the clinical application of EFE, the results of a repeated-dose toxicity test of EFE in mice demonstrated that EFE has low toxicity [12]. Prior to this clinical trial, a preliminary safety trial was performed in 7 healthy adult subjects. An EFE dose equivalent to an infusion of 6 g of Ephedra Herb was orally administered daily to the subjects for 2 weeks. An increase in the white blood cell count was observed in two of the subjects, but the causal relationship between EFE administration and increased white blood cell count remains unclear (unpublished data: UMIN000013176). Therefore, this clinical trial was aimed at investigating whether EFE is inferior to EHE in terms of safety when administered to humans.

## 2. Methods

### 2.1. Study Drug

#### 2.1.1. Study Drug Preparation Method

EFE and EHE were prepared according to Good Manufacturing Practice (GMP) standards. EFE was prepared as follows: EHE was extracted with hot water from Japanese Pharmacopoeia-grade Ephedra Herb (*Ephedra sinica *Stapf.). The EAs were then eliminated from EHE by using cation exchange column chromatography (DIAION SK1B resin, Mitsubishi Chemical Co.). Eph and Pse were confirmed to be undetectable (limit of detection: 10 ppm) in the extract. The resulting product was the EFE bulk extract [11]. After sterilizing the bulk extract at 90°C for 60 min, Japanese Pharmacopoeia-grade dextrin was added as the excipient, and the mixture was freeze-dried to obtain EFE (study drug).

EHE was prepared as follows: EHE was extracted with hot water from Japanese Pharmacopoeia-grade Ephedra Herb (*Ephedra sinica* Stapf.) to produce the bulk extract, which had an Eph concentration of 1.32% and a Pse concentration of 0.54%. After sterilizing the bulk extract at 90°C for 60 min, Japanese Pharmacopoeia-grade dextrin was added as the excipient, and the mixture was freeze-dried to produce EHE (control drug).

#### 2.1.2. Drug Administration Method

The daily EHE dose was equivalent to the amount extracted with hot water from 6 g of Ephedra Herb (crude drug). The daily dose of EFE was equivalent to the amount extracted with hot water from 6 g of Ephedra Herb (crude drug) with the EAs eliminated. The dosage was based on the fact that 6 g of Ephedra Herb is prescribed as an ingredient in eppikajutsuto, a Kampo formulation approved as a therapeutic drug (http://mpdb.nibiohn.go.jp/stork/). This is the maximum amount of Ephedra Herb contained in a Kampo formulation approved for therapeutic use. As Kampo formulations containing EFE instead of Ephedra Herb may be approved as therapeutic drugs in the future, this clinical trial verified the safety of the maximum Ephedra Herb amount, 6 g, contained in Kampo medicines. The subjects received the study drug twice daily, once in the morning and once in the evening (2 h after meals), in the form of 1 packet on each occasion (equivalent to the amount extracted with hot water from 3 g of Ephedra Herb) with water.

### 2.2. Recruitment of Subjects

Thirty-four candidates were screened by the physician-in-charge approved by Institutional Review Board (IRB). The screening was based on the following selection and elimination criteria. The selection criteria were as follows: (1) male sex; (2) age between 20 and 45 years; (3) voluntary consent for participation; (4) good health; (5) weigh of 50 kg or more but less than 100 kg, with BMI of 18 kg/m^2^ or more but less than 27 kg/m^2^; and (6) absence of any health problems, based on tests including medical examinations, vital signs, clinical test values, and electrocardiogram findings. The elimination criteria were as follows: (1) respiratory, digestive, cardiovascular, renal, hematological, mental, and neurological diseases, malignant tumors, or a history thereof; (2) prior digestive tract or renal surgery; (3) infectious diseases; (4) hypertension, hyperthyroidism, urinary disorders, or a history thereof; (5) a history of epileptic seizures or brain disorders, or likelihood of epileptic seizures; (6) food or drug allergies, or with a history thereof; (7) pollen allergies or allergic rhinitis; (8) smoking; (9) positive test findings for HBsAg, HCV, HIV, or syphilis; (10) positive urine drug tests or alcohol dependence; (11) receipt of therapeutic drugs within 4 weeks before the initial drug administration in Stage 1 of the clinical trial; (12) participation in clinical trials within 12 weeks of the start of drug administration in Stage 1 of the clinical trial; (13) donation of 200 ml of blood or more within 4 weeks or 400 ml of blood or more within 12 weeks before the start of the trial; and (14) ineligibility for participation in the clinical trial deemed by the physician-in-charge.

Thus, 25 candidates were deemed eligible for the clinical trial, out of whom 12 were finally selected to be the subjects of the clinical trial. Each subject was given a description of the clinical trial, conforming to a written description approved by Kitasato University's IRB. Each subject then voluntarily signed a consent form for participation in the clinical trial. Subsequently, the subjects were randomly allocated in a 1:1 ratio into 2 groups: prior administration of EFE (EFE-P) group and prior administration of EHE (EHE-P) group.

### 2.3. Study Design

This clinical trial was designed and performed as a single-institution, double-blinded, randomized, two-drug, two-stage, and comparative crossover study. This clinical trial conformed with the Good Clinical Practice (GCP) standards and was conducted in accordance with the protocol approved by Kitasato University's IRB.

### 2.4. Randomization and Blinding

The subjects were allocated to the EFE-P group or the EHE-P group in a 1:1 ratio by simple randomization, using the KiRS software developed by the Kitasato Clinical Research Center, Kitasato University School of Medicine. The trial staff responsible for allocating the study drugs prepared a chart according to the subject group and allocated the study drugs, whose external appearance was identical so as to render them indistinguishable. The allocation chart was then immediately sealed in an envelope to preserve the blinded nature of the trial. The blinding was maintained among all subjects, medical staff, and data sampling/management staff throughout the clinical trial period

### 2.5. Study Schedule

**Figure 1:**
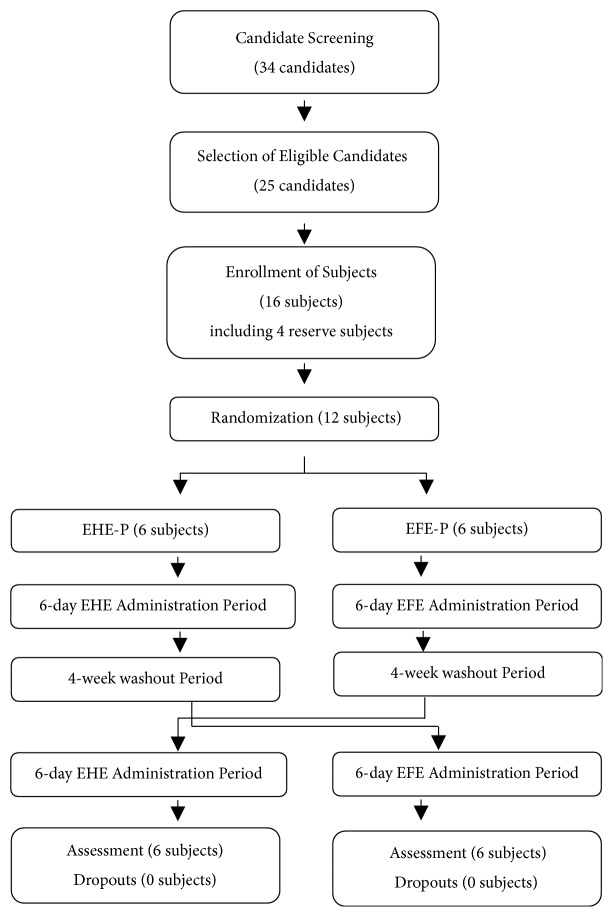
Clinical trial flowchart. Single-institutional, double-blinded, randomized, two-drug, two-stage, and comparative crossover study design and implementation (enrollment, allocation, follow-up, and data analysis of the subjects administered EHE and EFE in two stages). EHE-P: prior administration of EHE; EFE-P: prior administration of EFE group.

The trial was conducted in a hospital setting. In Stage 1, EFE and EHE were orally administered to the EFE-P and EHE-P groups, respectively, for 6 days. After a 4-week washout period, Stage 2 was started with a drug different from that administered in Stage 1; the drugs were orally administered for 6 days (Figure 1).

Table 1 shows the trial implementation schedule. At the start of the trial, in the first period, the subjects underwent a medical examination (subjective symptoms/physician's observations) conducted by the physician-in-charge, vital signs check (blood pressure, pulse rate, and body temperature), blood test, urinalysis, and electrocardiography; subjects' physical measurements (weight) were also recorded. Each day during the study drug administration period, the subjects underwent a medical examination, vital signs check, blood test, urinalysis, and electrocardiography, and their physical measurements were recorded. There was a 4-week washout period between Stage 1 and Stage 2. In Stage 2, the evaluation was performed per a similar schedule as that for Stage 1. Two weeks after Stage 1, and 1 week after Stage 2, the subjects underwent a medical examination conducted by the physician-in-charge, vital signs check, blood test, urinalysis, and electrocardiography, and their physical measurements were recorded.

### 2.6. End Points

The end-point assessment was mainly focused on any adverse events indicated by the results of the medical examination, blood test, blood biochemistry test, urinalysis, and electrocardiography. The severity of the adverse events was evaluated according to the Common Terminology Criteria for Adverse Events (CTCAE) v4.0: https://ctep.cancer.gov/protocoldevelopment/electronic_applications/ctc.htm). We regarded Grade 1 in CTCAE classification as mild; Grade 2 as moderate; and Grade 3 or high as severe. With regard to the causal relationship with the study drug, “unrelated” means “when there is no temporal correlation, and it can be clearly demonstrated that other factors (current diseases, comorbidity, concomitant drugs, or concurrent procedures) are responsible.” “Probably unrelated” means “when there seems to be no temporal correlation, or there is a high probability that other factors are responsible.” “Possibly related” means “when there is a clear temporal relationship, and other factors can be presumed to be responsible, but the possible role of the study drug cannot be dismissed (for instance, similar adverse events by the study drug or similar compounds have been previously reported, or causal relationship can be presumed by its pharmacological activity). “Probably related” means “when there is a temporal relationship, and almost all of the factors other than the study drug have been eliminated. “Clearly related” means “when there is a temporal relationship, and the pharmacological effect of the study drug can be easily explained.” Among these assessments, “probably related” and “clearly related” were considered indicators of a causal relationship between the study drug and the adverse event.

The total number of adverse events was determined. If the same subject experienced several different types of adverse events (for example, if the same subject experienced headaches and showed an increased white blood cell count and increased body temperature), all of them were counted (in the above example, 3 adverse events would be counted in total). However, if a subject experienced the same adverse event (for example, headaches) twice or more (headaches), it was only counted once. Meanwhile, the incidence of each adverse event for each study drug administered to the 12 subjects was evaluated based on the number of subjects who experienced the said adverse event.

### 2.7. Statistical Analysis

The number of subjects required for the trial was 12, six in the EHE-P group and six in the EFE-P group. This number was determined considering the minimum number of subjects necessary for a safety evaluation, the commitments required from the subjects, and the feasibility of a crossover study. The incidence of adverse events during EFE administration was compared with that during EHE administration. Data were analyzed using Fisher's exact test. The differences between the 2 groups at a significance level of 5% were determined. Furthermore, the odds ratio for the adverse events and the 95% confidence intervals were determined. JMP pro13 (SAS Institute Inc.) software was used for analysis.

## 3. Results

### 3.1. Subjects

For this clinical trial, 34 candidates were screened, out of whom 25 were deemed eligible. A preliminary group of 16 subjects, including 4 reserve subjects, was formed. Finally, the group was narrowed down to 12 subjects. Between the EHE-P group (6 subjects) and the EFE-P group (6 subjects), there were no significant differences in variables such as age, height, weight, vital signs, and blood test results. (Table 2). All subjects complied with instructions on ingesting the study drugs and completed the schedule for examinations. No subjects dropped out during the trial.

### 3.2. Adverse Events

Twenty-five cases of adverse events were noted during the course of the entire trial. However, all adverse events were “mild” and did not have any detrimental effects on the affected subjects' daily activities. In 11 cases, the adverse events showed a causal relationship with the study drugs. The most frequent adverse event was increased pulse rate (5 cases), followed by insomnia (4 cases). The remaining adverse events were hot flashes and dysuria (1 case each). Eight adverse events were attributable to EHE administration and three to EFE administration.

When the incidence of each adverse event was classified according to the cause, EHE administration and EFE administration accounted for increased pulse rate in 4 cases and 1 case, respectively (p=0.32). Further, EHE administration and EFE administration accounted for insomnia in 3 cases and 1 case, respectively (p=0.59). There was only one case of hot flashes due to EFE administration (p=1.00) and one case of dysuria due to EHE administration (p=1.00). No significant difference in the incidence of each adverse event was observed between EHE administration and EFE administration. The odds ratios for the adverse events also showed no significant results (Table 3).

## 4. Discussion

In this clinical trial, no adverse events worse than “weak in severity” were observed for both EFE administration and EHE administration. Among a total of 24 subjects, there were 25 adverse events, all of which were mild, and 11 instances of these had a causal relationship with the study drugs. The adverse events were increased pulse rate, insomnia, hot flashes, and dysuria, all of which have been previously recognized as side effects of EAs. However, even after EAs had been eliminated, there was one case each of increased pulse rate, insomnia, and hot flashes during EFE administration. These might be attributable to substances other than EAs or a nocebo effect.

When the incidence of each adverse event was examined, in the case of EHE administration, increased pulse rate was noted in 33%, insomnia in 25%, and dysuria in 8% of the subjects. To our knowledge, no previous surveys of the incidence of adverse events after EHE administration have been reported, and we believe this to be the first such report. The subjects in this clinical trial were relatively young (age range, 20-45 years, average: 35.4 years). Because an increase in the incidence of adverse events would be expected for an older sample population, caution is required when Ephedra Herb is used by individuals older than those in this study. On the other hand, it was observed that the incidences of increased pulse rate, insomnia, and dysuria associated with EFE administration (8%, 8%, and 0%, respectively) were lower than those associated with EHE administration. Moreover, the total number of adverse events was 8 in the case of EHE administration and 3 in the case of EFE administration.

Recently, we investigated the adverse events after a single application of EHE or EFE in mice. When a dose of 700 mg/kg EHE was orally administered to mice, excitation, insomnia, and arrhythmias were observed. However, these adverse events were not observed when EFE was orally administered to the mice at the same dose [13]. This observation suggests that adverse events caused by EAs do not occur when EFE is used. The empirical results observed in mice ingesting far greater doses than those administered clinically are considered to denote the latent risks of EAs. It is, therefore, reasonable to infer that EFE is safe. The results of this trial closely reflect this view; however, there was no significant difference in the incidences of adverse events between EHE and EFE. One reason for this might be the small sample size, as this trial was designed only to verify the noninferiority of EFE to EHE in terms of safety. In the future, a larger sample would be needed to establish whether EFE is superior with regard to safety.

As the increased white blood cell count due to EFE noted in the preliminary trial was not observed in both the EFE and EHE groups in this clinical trial, no causal relationship with the administration of the study drugs could be inferred. Because there have been no previous reports of increased white blood cell count due to EHE ingredients, including EAs, the appearance of this condition in the preliminary trial suggests that the subjects involved might have had a slight infection that coincidentally affected the results.

As this study was the first full-fledged investigation of EFE safety in humans, it was necessary to restrict the subjects to younger men. However, since patients in actual clinical settings include older persons and female patients, future studies on EFE safety will need to investigate EFE's safety for these patients as well. Further, the study drug administration period of 6 days in this clinical trial was relatively short. Because joint pain and suppression of metastatic cancer may be included in the scope of future EFE clinical applications, safety investigations in a longer administration period will be needed in the future.

In conclusion, the results of this clinical trial clearly demonstrated that EFE is not inferior to EHE in terms of safety. The next step toward the clinical application of EFE will be clinical research to establish EFE's efficacy against pain, influenza virus infection, and metastatic cancer.

## Figures and Tables

**Table 1:** Complete trial schedule for subjects, including preliminary assessment, stage 1, assessment during washout period, stage 2, and post-administration assessment.

Stage	Preliminary Assessment	Stage 1							Assessment during Washout Period	Stage 2						Post-administration Assessment
Protocol Activities	Day -26~-1	Day 0	Day 1	Day 2	Day 3	Days 4~6	Day 7	Day 8	Day 22	Day 35	Day 36	Day 37	Days 38~40	Day 41	Day 42	Day 49

Inclusion/Exclusion Decision	◯															

Acquisition of Consent	◯															

Hospitalization		◯	◯	◯	◯	◯	◯	◯		◯	◯	◯	◯	◯	◯	

Administration of Study Drugs				◯	◯	◯	◯				◯	◯	◯	◯		

Medical Examination by Physician	◯	◯	◯	◯	◯	◯	◯	◯	◯	◯	◯	◯	◯	◯	◯	◯

Blood Pressure/Pulse Measurement	◯	◯		◯	◯	◯	◯	◯	◯	◯	◯	◯	◯	◯	◯	◯

Body Temperature Measurement	◯	◯		◯	◯	◯	◯	◯	◯	◯	◯	◯	◯	◯	◯	◯

Weight Measurement	◯	◯						◯	◯	◯					◯	◯

Blood Test	◯	◯		◯	◯	◯	◯	◯	◯	◯	◯	◯	◯	◯	◯	◯

Urinalysis	◯	◯		◯	◯		◯	◯	◯	◯	◯	◯		◯	◯	◯

Electrocardiogram	◯	◯		◯	◯		◯	◯	◯	◯	◯	◯		◯	◯	◯

**Table 2:** Baseline characteristics of subjects, vital signs, and hematological findings.

n	All Subjects	EHE-P	EFE-P
	12	6	6
Age, years	35.4±7.9	36.5±9.7	34.3±6.5
Height, cm	172.7±5.0	174.1±6.1	171.3±3.5
Weight, kg	62.2±5.3	61.2±6.9	63.3±3.3
Body temperature, °C	36.5±0.3	36.5±0.3	36.5±0.3
Systolic blood pressure, mmHg	113.7±6.9	111.8±7.4	115.5±6.5
Diastolic blood pressure, mmHg	72.5±7.7	70.8±8.5	74.2±7.2
Pulse rate, /min	70.7±10.6	71.8±10.5	69.5±11.6
White blood cell count, /× 1000 *μ*L	4.1±1.0	4.5±1.0	3.6±0.8
Hemoglobin level, g/dl	14.1±0.7	13.9±0.7	14.4±0.6
Blood platelet count, ×10^3^/*μ*L	227±55	238±68	217±42

EHE-P: prior administration of EHE group; EFE-P: prior administration of EFE group

**Table 3:** Comparison of various adverse events during EFE and EHE administration. p value derived from Fisher's exact test.

	Incidence of Adverse Events			Odds ratio (confidence interval) of incidence during EFE administration/during EHE administration
	During EFE administration	During EHE administration	p value	
Increased pulse rate	1 case/12 cases	4 cases/12 cases	0.32	0.18 (0.01-1.52)
Insomnia	1 case/12 cases	3 cases/12 cases	0.59	0.27 (0.01-2.56)
Hot flash	1 case/12 cases	0 cases/12 cases	1.00	not applicable
Dysuria	0 cases/12 cases	1 case/12 cases	1.00	not applicable

## Data Availability

The data used to support the findings of this study are available from the corresponding author upon request.
